# Unveiling the Anticancer Potential of a New Ciprofloxacin-Chalcone Hybrid as an Inhibitor of Topoisomerases I & II and Apoptotic Inducer

**DOI:** 10.3390/molecules29225382

**Published:** 2024-11-15

**Authors:** Doaa Mohamed Elroby Ali, Hossameldin A. Aziz, Stefan Bräse, Areej Al Bahir, Abdullah Alkhammash, Gamal El-Din A. Abuo-Rahma, Ali M. Elshamsy, Hamada Hashem, Walid M. Abdelmagid

**Affiliations:** 1 Department of Biochemistry, Faculty of Pharmacy, Sohag University, Sohag 82524, Egypt; 2 Department of Pharmaceutical Chemistry, Faculty of Pharmacy, New Valley University, New Valley 72511, Egypt; 3 Institute of Biological and Chemical Systems—Functional Molecular Systems (IBCS-FMS), Karlsruhe Institute of Technology (KIT), Kaiserstrasse 12, 76131 Karlsruhe, Germany; 4 Chemistry Department, Faculty of Science, King Khalid University, Abha 64734, Saudi Arabia; 5 Department of Pharmacology, College of Pharmacy, Shaqra University, Shaqra 11961, Saudi Arabia; 6 Department of Medicinal Chemistry, Faculty of Pharmacy, Minia University, Minia 61519, Egypt; 7 Department of Pharmaceutical Chemistry, Faculty of Pharmacy, Deraya University, New Minia City 61768, Egypt; 8 Department of Pharmaceutical Chemistry, Faculty of Pharmacy, Sohag University, Sohag 82524, Egypt; 9 Medicinal Chemistry and Drug Discovery Research Centre, Swenam College, 210-6125 Sussex Avenue, Burnaby, BC V5H 4G1, Canada; wabdelmagid@swenamcollege.ca

**Keywords:** ciprofloxacin, anti-proliferative, topoisomerases I & II inhibitor, apoptotic inducer, molecular docking

## Abstract

The current study has yielded promising results in the evaluation of a new ciprofloxacin-chalcone hybrid (CP derivative) for its anticancer activity as potential Topoisomerases (Topo) I and II inhibitors. The in vitro results showed that the CP derivative significantly suppressed the growth of HCT-116 and LOX IMVI cells, with IC_50_ values of 5.0 μM and 1.3 μM, respectively, outperforming Staurosporine, which had IC_50_ values of 8.4 μM and 1.6 μM, respectively. Flow cytometry analysis revealed that the new CP derivative triggered apoptosis and cell cycle arrest at the G2/M phase, associated with the up-regulation of pro-apoptotic genes (Bax and Caspase 9) and downregulation of the anti-apoptotic gene (Bcl-2). Further investigations showed that the CP derivative inhibited Topo I and II enzymes, as expected molecular targets; docking studies further supported its dual inhibitory action on Topo I and II. These findings suggest that the ciprofloxacin-chalcone hybrid could be a promising lead compound for developing new anticancer therapy.

## 1. Introduction

Worldwide, cancer is a serious health problem [1]. More than 150 different diseases are classified as cancer on a cellular level, with increased cell proliferation and downregulation of pro-apoptotic pathways, making cancer one of the leading causes of death globally [2]. In 2020, there were 10 million cancer-related deaths worldwide out of the 19.3 million newly diagnosed cases of the disease [3]. Current cancer treatments frequently cause cytotoxicity and genotoxicity [4]. The primary challenges in cancer management are the lack of specificity and significant side effects of the present cancer treatment strategies [5]. The global pursuit to uncover new anticancer therapies is fueled by the inadequacies of current cancer treatment [6].

Ciprofloxacin (CP) is a widely used second-generation fluoroquinolone antibiotic for treating human bacterial infections. CP exerts its antibacterial effects by blocking the enzyme of the bacterial DNA gyrase (topoisomerase II) [7]. Bacterial DNA topoisomerases play a crucial role in controlling and altering DNA structure during various cellular processes such as cell differentiation, proliferation, and survival [8]. Recent research studies have shown that CP has non-classical biological activity, acting as a cytotoxic agent and inducing apoptosis [9]. Several ciprofloxacin derivatives have demonstrated their anticancer effects by targeting several molecular pathways, including the inhibition of Topos I/II, [10] induction of apoptosis, and cell cycle arrest [1]. CP is considered a versatile compound that serves as a basis for structural modifications in the advancement of innovative anti-cancer medications [11]. It was reported that introducing a substituent at position 4 of the piperazinyl moiety at C-7 in the CP framework substantially influences its physicochemical characteristics, biological activity, and its ability to inhibit mammalian DNA Topos I/II [7]. Consequently, numerous C-7 modified CP compounds exhibited a wide range of cytotoxic effects, specifically targeting tumor cells with great selectivity [9].

Moreover, chalcones are a fundamental component of numerous anticancer agents. Chalcone-containing compounds, which are cytotoxic agents, exert their effects by activating various molecular pathways [12]. These pathways include triggering the apoptotic cascade by increasing the expression of pro-apoptotic proteins like Caspase 9 and Bax while reducing the expression of anti-apoptotic proteins like Bcl-2. Additionally, these compounds inhibit tubulin polymerization and proliferation [13].

**Figure 1:**
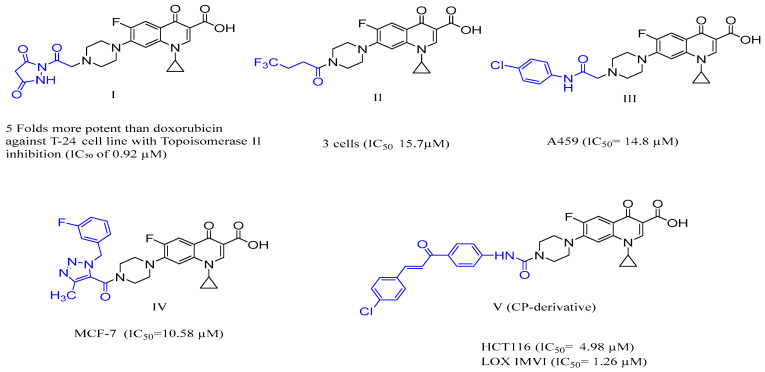
Structure of various N-4 piperazinyl-modified CP compounds with anticancer activity, including target compound **V** (CP derivative 2).

As a result, various hybrids of ciprofloxacin were prepared and evaluated for their anticancer activities, including compound **I,** which is an N-4-piperazinyl-dioxopyrazolidine derivative of ciprofloxacin and showed more than five times more potency than the doxorubicin toward the T-24 cell line via Topo **II** inhibition with an IC_50_ value of 0.92 µM [14]. Also, compound **II** showed higher activity against prostate PC3 cancer cells with IC_50_ equal to 15.7 µM [15]. Moreover, compound **III** exhibited remarkable and selective antitumor activity toward non-small cell lung cancer with an IC_50_ value of 14.8 μM [16]. Furthermore, compound **IV** showed anti-cancer efficacy against MCF-7 with IC_50_ = 10.58 µM (Figure 1) [17].

From that point onwards, researchers initiated contemplation on discovering more derivatives of Cp to get the required outcomes in its superiority over its main counterpart derived from it. Hamada et al. have recently synthesized novel urea-linked CP-chalcone hybrids as anticancer agents, aiming to exaggerate the weak anticancer of the parent ciprofloxacin. Among these synthesized compounds was compound **V** [18], gathering CP nucleus, chalcone, and urea moiety, which is commonly utilized in drug design methods due to their ability to generate several stable hydrogen bonds with target proteins [19] in one compact compound that has the empirical formula C_33_H_28_Cl FN_4_O_5_. This study aimed to examine the inhibitory effects of compound **V** on cell growth and induction of cell death in the HCT116 colorectal cancer cell line. Additionally, the study aimed to assess the compound’s ability to halt the cell cycle and promote apoptosis through the caspase 9, Bax, and Bcl-2-dependent pathways.

## 2. Results

### 2.1. Chemistry

**Scheme 1:**
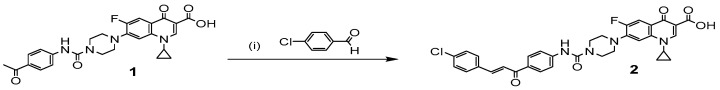
Synthesis of the CP derivative 2. **Reagents and conditions:** (i) 60% NaOH, ethanol, 0–5 °C stirring overnight.

CP derivative 2 was prepared as previously reported [7,18] by condensation of CP derivative 1 with *p*-chlorobenzaldehyde in ethanol in the presence of a 60% sodium hydroxide solution to afford the target compound in a good yield, Scheme 1.

### 2.2. Biology

#### 2.2.1. In Vitro Screening of the Anticancer Activity at a Single Dose of 10 μM

**Figure 2:**
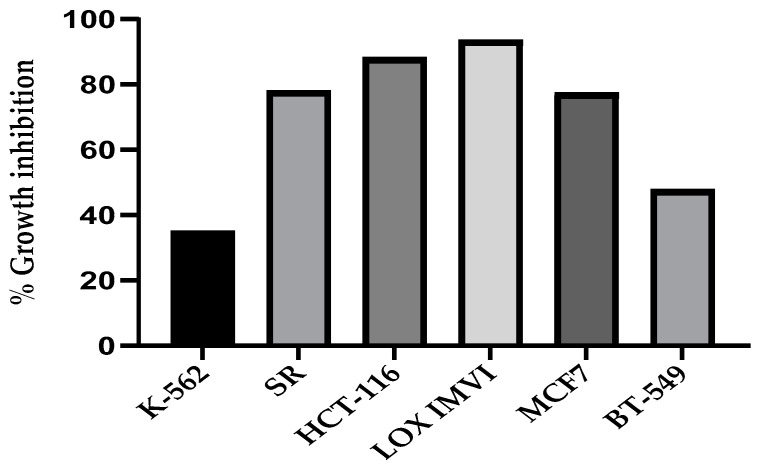
Growth Inhibition Percentages of CP Derivative against Cancer Cell Lines (K-562, SR, HCT-116, LOX IMVI, MCF7, and BT-549) at a dose of 10 μM single screening.

According to NCI protocols, the target compound CP derivative was screened for its anticancer activity in NCI, USA, against sixty cancer cell lines [16]. As shown in Figure 2, the results of the single dose of 10 µM showed that the CP derivative exhibited remarkable growth inhibition against various cancer cell lines, especially against SR (leukemia), HCT-116 (colon), LOX IMVI (melanoma), and MCF7 (breast) cancer cells with growth inhibition percents of 78.0, 88.2, 93.5, and 77.4, respectively. These results inspire us to determine the IC_50_ of this compound against LOX IMVI, HCT-116, and WI-38 normal cells and to explore its mechanism of action as a potential anticancer lead.

#### 2.2.2. Cell Viability Assay

**Figure 3:**
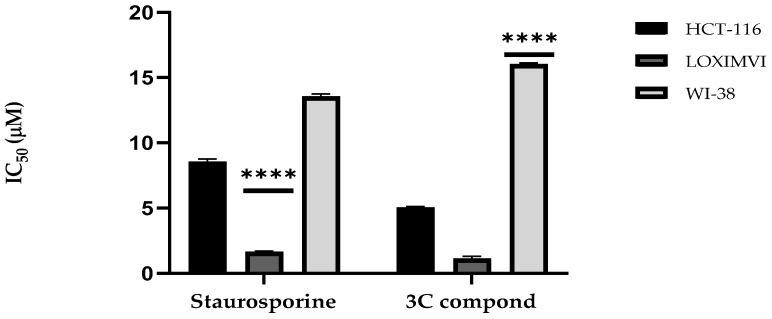
Comparative IC_50_ (µM) Values of CP Derivative and Staurosporine (Positive Control) against Cancer Cell Lines (HCT-116 and LOX IMVI) and Normal Cell Line (WI-38). Bars display the mean ± SD. Statistical analysis via Two-way ANOVA and the Tukey–Kramer test afterward reveals significant differences (**** *p* < 0.0001) relative to Staurosporine.

The effect of the CP derivative on the survival of colon HTC-116 and melanoma LOX IMVI cancer cells following incubation for 24 h. Results showed that the CP derivative exhibited higher potency than the positive control Staurosporine against HCT-116 cancer cells, with IC_50_ equals 5.0 μM and 8.4 μM, respectively. Also, CP derivative showed more potent activity against LOX IMVI cancer cells than Staurosporine with IC_50_ values of 1.3 μM and 1.6 μM, respectively. Moreover, the cytotoxic effect of the CP derivative on normal cell WI 38 was investigated compared to Staurosporine as a positive control. Results revealed a lower potency of CP derivative with IC_50_ equal to 16.0 μM compared to Staurosporine with IC_50_ equal to 13.3 μM, as shown in Figure 3. These results mean that the target compound is more potent against the tested cancer cell lines and less toxic toward normal cells, which can serve as a lead compound for further optimization to develop novel anticancer drugs.

#### 2.2.3. Annexin V Assay Using Flowcytometry

**Figure 4:**
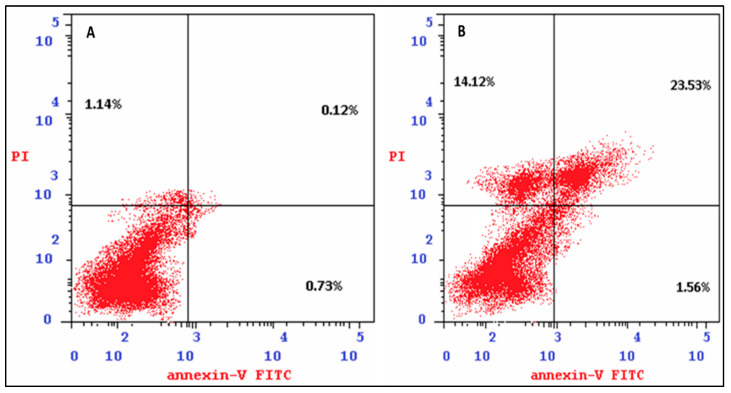
The apoptosis and necrosis assay of colon HCT-116 induced by DMSO (control, (**A**)) and CP derivative (IC_50_, 5 µM, (**B**)).

**Figure 5:**
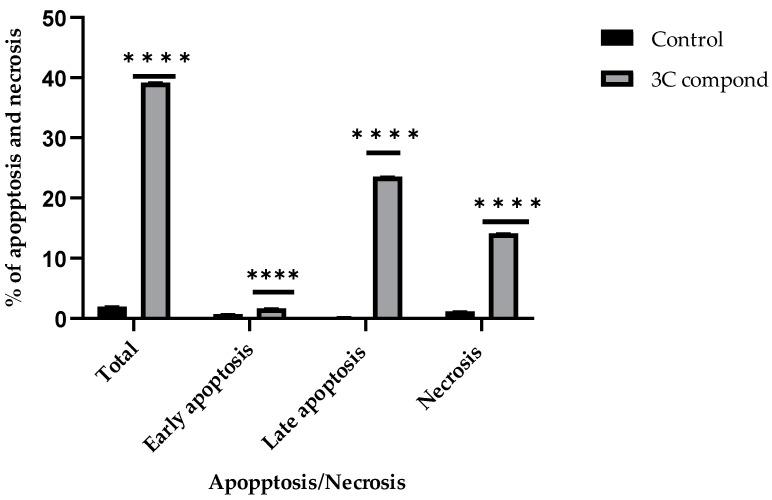
The percentages of early apoptosis, late apoptosis, total apoptosis, and necrosis induced by the IC_50_ concentration of the CP derivative in comparison to the untreated control on HCT-116 colon cancer cells. Bars represent the mean ± SD. Statistical significance was determined using a two-way ANOVA test and the Tukey–Kramer test afterward, with **** *p* < 0.0001 indicating a significant difference compared to the control group.

The apoptotic ability of the CP derivative was studied to decide whether its anticancer activity against HTC-116 cancer cells is associated with increased apoptosis and necrosis. Cell apoptosis and necrosis were estimated using an Annexin V assay. Exposure of HCT-116 cells to the IC_50_ concentration of the examined compound for 24 h induced apoptosis and necrosis. The proportion of early apoptotic (Annexin V-positive/PI-negative) cells significantly (*p* < 0.001) increased from 0.73% (untreated cells) to 1.56% (treated cells). It also significantly increased the percentage of late apoptotic (Annexin V-positive/PI-positive) cells from 0.12 (untreated cells) to 23.53 (treated cells) in HCT116 (*p* < 0.001). Furthermore, it significantly increased the percentage of the necrosis (Annexin V-positive/PI-positive) cells from 1.14 (untreated cells) to 14.12 (treated cells) in HCT116 (*p* < 0.001) (Figure 4 and Figure 5). As a result, induction of apoptosis and necrosis triggers the anticancer of the CP derivative.

#### 2.2.4. Cell Cycle Analysis Using Flowcytometry

**Figure 6:**
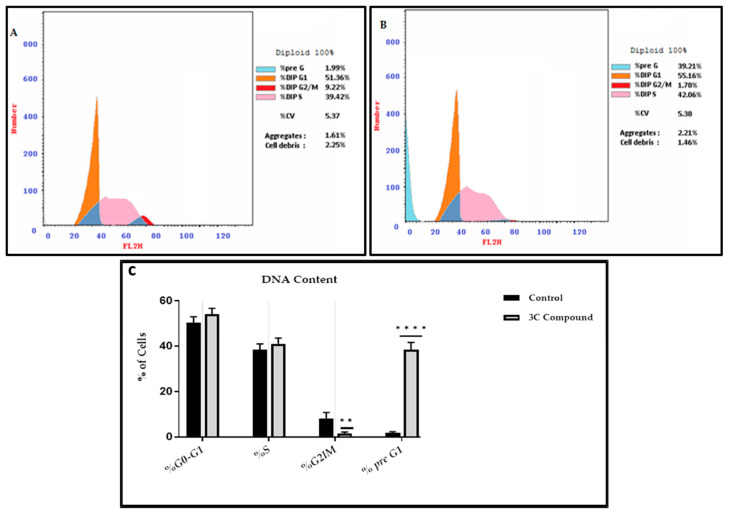
Flow cytometric analysis illustrating the distribution of cell cycle phases in HCT-116 cells, comparing untreated controls and cells treated with the IC_50_ concentration of the CP derivative for 24 h. (**A**) Dot plots representing the cell cycle phases for untreated cells after PI staining and (**B**) treated cells under the same conditions. (**C**) Quantitative comparison of cell proportions in each phase (G0/G1, S, G2/M, and pre-G1) between untreated and treated groups. Data are shown as mean ± SD. A two-way ANOVA and the Tukey–Kramer test afterward were used for statistical analysis, with ** *p* < 0.01 and **** *p* < 0.0001 indicating significant differences compared to untreated cells.

Cell growth is primarily governed by cell cycle regulatory systems [20]. Cancer cells can be prevented from multiplying by causing them to stop progressing through the cell cycle [21]. The G2/M checkpoint in the cell cycle is a potential target for cancer therapy [22]. The CP derivative was chosen to investigate its impact on the course of the cell cycle of HCT-116 cells. The administration of HCT-116 cells with the CP derivative disrupted their regular cell cycle distribution. The findings were disclosed. The CP derivative caused a 20-fold rise in the proportion of cells at the pre-G1 stage while causing an 80.7% drop in DNA content at the G2/M stages (Figure 6). The presence of sub-G1 peaks in the cell cycle profile confirms the accumulation of cells in the pre-G1 phase, indicating a potential role of apoptosis. According to these findings, the CP derivative caused a halt in the cell cycle at the G2-M phase, which resulted in the suppression of cell growth and the initiation of programmed cell death. These results are consistent with earlier research showing that fluoroquinolones cause G2/M arrest and trigger apoptosis in cancer cells [23].

#### 2.2.5. Expression of Bax, Bcl-2, and Caspase 9 Genes

**Figure 7:**
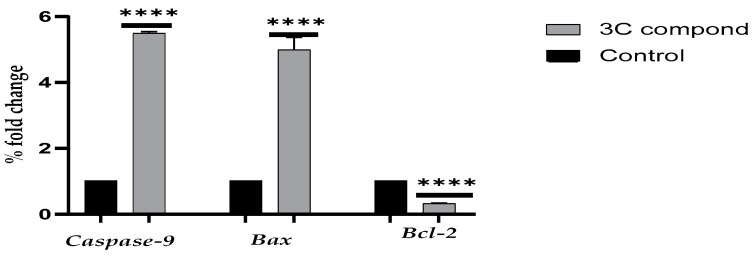
Quantitative real-time PCR analysis of caspase-9 Bax and Bcl-2 expression levels in HCT-116 cells following 24 h treatment with the IC50 concentration of the drug, normalized to β-actin. Bars indicate mean ± SE. Statistical significance was assessed using an unpaired *t* test, with **** *p* < 0.0001 compared to untreated cells (control).

In this study, Bax, Bc-l2, and Caspase 9 genes were amplified and normalized to β-actin using quantitative real-time PCR, as shown in Figure 7. Compared to the untreated cells, the Bax and Caspase 9 gene expression significantly (*p* < 0.001) increased by 4.61 and 5.57. fold in HCT-116 cells treated with CP derivative, respectively. While BCl-2 expression was significantly (*p* < 0.001) decreased in HCT-116 cells by 0.32 compared to untreated cells.

#### 2.2.6. Inhibitory Action of CP Derivative on Topoisomerase I/II

For the CP derivative, the inhibitory effect on Topo I and Topo II was assessed using gel electrophoresis technique according to the reported protocols [16]. As a control, topotecan, a known inhibitor of the Topo I enzyme [18], was utilized. Etoposide, a recognized inhibitor of Topo II, [24] was also employed as a control. Table 1 shows the findings of Topo I and II. The CP derivative inhibited Topo I less effectively than topotecan (IC_50_ = 37.5 and 23.0 µM, respectively), while it inhibited Topo II with an IC_50_ of 19.92 µM more potent than the reference compound etoposide with an IC_50_ of 28.9 µM. These findings demonstrate that the target compound has dual inhibitory action against Topo I/II, implying its anti-proliferative activity, as shown in Table 1.

### 2.3. In Silico Studies

#### 2.3.1. Docking Studies

The newly synthesized CP derivative in comparison to topotecan was theoretically studied via docking into the active pocket of Topo I enzyme (PDB: 1K4T) [25] and Topo II (PDB: 7YQ8) [26] to spot the potential binding interactions induced by the new structural alterations to ciprofloxacin using topotecan as a positive control for Topo I and etoposide as a positive control for Topo II. The ligands in PDB files were redocked into the active site to validate the docking methodology. The poses obtained were almost identical to the original binding patterns, as shown in Figures S7 and S8. The validity of the docking process and its ability to accurately predict the right orientation of the CP derivative is supported by the values of the root-mean-square deviation (RMSD) of the redocked co-crystallized ligands being less than 1.5 Å. Negative binding scores ensure that fluoroquinolone derivatives spontaneously bind to the active sites of the Topo I and II enzymes.

**Figure 8:**
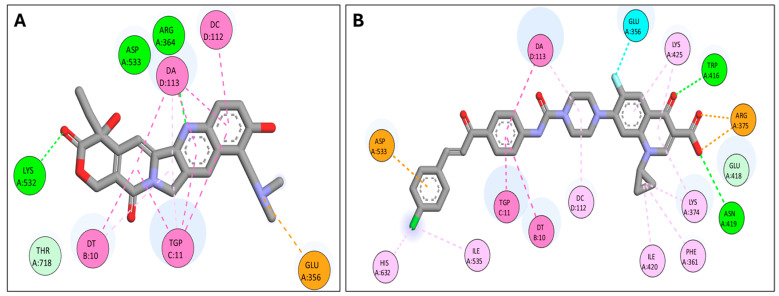
Two-dimensional interactions in Topo I active site (PDB: 1K4T); (**A**) Topotecan binding interactions; (**B**) CP derivative binding interactions.

**Figure 9:**
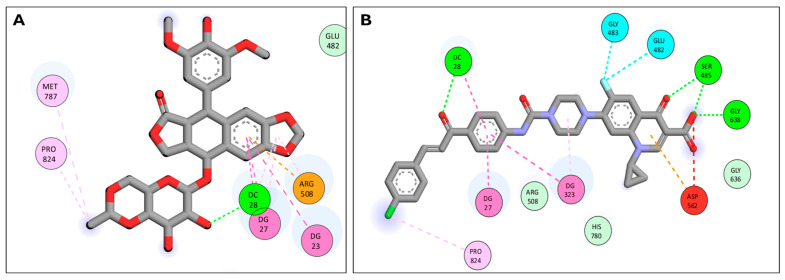
Two-dimensional interactions in Topo IIβ active site (PDB: 7YQ8); (**A**) Etoposide binding interactions; (**B**) CP derivative binding interactions.

The docked CP derivative has high affinity for the Topo I and II enzymes with binding free energy (∆G) values of −12.04 and −8.01, comparable to topotecan affinity to Topo I and etoposide affinity to Topo II with binding free energy (∆G) values of −10.26 and −8.01, respectively, as shown in Table 2. CP derivative showed good binding with the active site of Topo I (PDB: 1K4T) via hydrogen bonding with amino acid residues LYS 532, ASP 533, and ARG 364, in addition to binding with nucleotide bases DT 10, DA 113, and DG 112 via Pi-Pi interactions, Figure 8. Also, CP derivative showed binding with the active site of Topoisomerase II (PDB: 7YQ8) by hydrogen bonding and Pi-alkyl interactions with the chalcone part, Figure 9. Importantly, the docking study of the CP derivative with the active sites of Topo I/Topo II goes ahead with its anticancer activity and its inhibition of topoisomerase enzymes.

#### 2.3.2. Predictions of Physicochemical and Pharmacokinetic Properties

**Figure 10:**
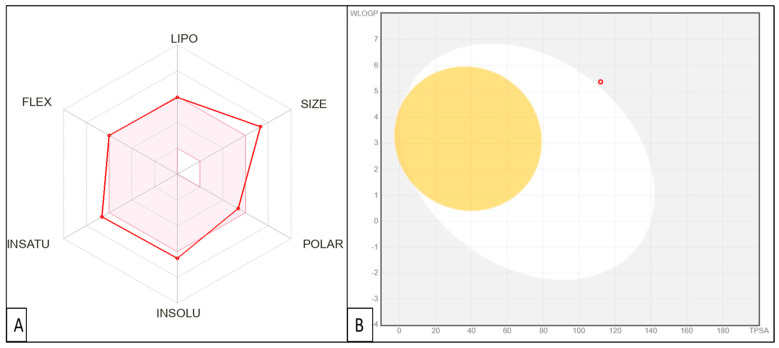
(**A**) Rader model for CP derivative; (**B**) The BOILED-Egg model of CP derivative.

Potential drug candidates must exhibit a reasonable pharmacokinetic profile to reach the clinic. Consequently, the physicochemical and pharmacokinetic characteristics of CP derivative were forecasted via Swiss ADME, as illustrated in Table 3 and Figure 10; CP derivative was predicted to have poor gastrointestinal absorption and not to cause centrally adverse effects as it is predicted not to pass the blood–brain barrier. CP derivative was predicted to be resistant to P-gp efflux. The expected impact of the target drug on CYP450 enzymes, namely CYP1A2, CYP2D6, and CYP3A4, suggests a low likelihood of drug–drug interactions occurring. CP derivatives were expected not to be inhibitors for all CYP enzymes except CYP2C9. Moreover, CP derivative has a molecular weight exceeding 500 g/mol, resulting in one Lipinski violation, with no violations in the Veber and Egan filters. Furthermore, the CP derivative has three violations in the Ghose filter (MW > 480, MR > 130, atom number > 70) and has one violation in Muegge (XLOGP3 < −2). The Abbott bioavailability score was 0.56.

The bioavailability radar included six physicochemical properties: lipophilicity, size, polarity, solubility, flexibility, and saturation. The lipophilicity of the CP derivative is in the acceptable range (XLOGP3 from −0.7 to +5.0), the polarity of the target compound is within a reasonable range (TPSA from 20 Å2 to 130 Å2), and the instauration degree is reasonable (Fraction Csp3 0.21). Finally, the target compound exhibited adequate flexibility (rotatable bonds equal to 9).

The BOILED-Egg approach is a reliable model that precisely predicts the permeation of substances in the gastrointestinal tract and the brain. It achieves this by calculating specific compounds’ lipophilicity (measured in terms of WLOGP) and polarity (measured in terms of TPSA). The target compound does not cross the blood–brain barrier, indicating its favorable safety profile for the central nervous system. Further structural modifications and optimizations are required to enhance the ADME properties of the CP derivative.

## 3. Discussion

In complicated diseases, such as malignancies, inflammation, neuropsychiatric disorders, and viral and bacterial infections, multitarget therapy has piqued the interest of biologists and medicinal chemists for many years [27]. Ciprofloxacin derivatives have been utilized as antibiotics for more than four decades, but they are now also being exploited as pharmacological substances with a wide range of applications [28]. Also, these derivatives have been repositioned as anti-cancer agents due to their potent pro-apoptotic, antiproliferative, immunomodulatory, and anti-metastatic properties [18]. Colorectal cancer (CRC) is one of the most commonly diagnosed cancers in both genders and is the fourth most deadly cancer worldwide after liver, lung, and stomach cancers [29]. NCI tested 60 cell lines originating from nine cancer cell lines, including colon, melanoma, leukemia, lung, prostate, CNS, ovarian, renal, and breast cancer cell lines, for the multitarget anticancer efficacy of the newly synthesized CP derivative. This anti-cancer effect could be mediated by decreasing the activity of DNA topoisomerase I and II in eukaryotes [24]. As a result, mitochondrial damage, electron transport chain blockage, and ATP depletion occur. Apoptosis is favored by energy depletion. The current study looked at the biological antiproliferative properties of the CP derivative against HCT-116 cells; it has the dual inhibitory activity of topoisomerase I and II. Positive controls for topoisomerases I and II were topotecan and etoposide (respectively). Compared to the positive control topotecan, the CP derivative demonstrated slightly lower potent inhibitor action against Topo I but more potent inhibitor activity against Topo II compared to the positive control etoposide. These findings support Hong-Xia Liang and colleagues’ findings that fluoroquinolone derivatives have a strong anti-cancer effect by decreasing DNA topoisomerase II activity and interfering with DNA replication in human hepatocellular carcinoma cells SMMC-7721 [30].

Apoptosis defects can result in aberrant proliferative cells, enhance malignant transformation, and increase chemotherapeutic treatment resistance [31]. Recently, research studies have demonstrated apoptosis and its role in the intervention of the lethal properties of antineoplastic agents in cancer cells. The antitumor activity of the CP derivative may be due to apoptosis or necrosis. As a result, compared to the non-treated control, treating HCT-116 cells with the CP derivative resulted in a significantly substantial rise in the proportion of apoptotic cells. A considerable increase in the number of necrotic cells was also seen when compared to the non-treated control. The overall number of early and late apoptotic cells outnumbers necrotic cells. These findings suggest that the CP derivative causes cell death via apoptosis in the HCT-116 cell line.

Further, we analyzed the cell cycle of the HCT-116 cells before and after treatment with the tested CP derivative. We discovered a significant increase in the pre-G1 peak and cell growth arrest in the G2/M phase—these findings agree with the previously reported data. Tumor cells may also limit apoptosis by molecular processes, such as downregulating proapoptotic genes like Bax and overexpressing antiapoptotic genes like Bcl-2 [32]. In addition, Caspases-9-induced apoptosis is a type of cell death triggered by this class of proteases that has been identified as an effective cancer therapeutic target [33]. To confirm that, we analyzed the levels of these different genes to understand the mechanism by which the CP derivative exerts its antitumor activity. Treatment of HCT-116 cells with the compound under investigation resulted in increased expression of Bax and Caspase 9 pro-apoptotic genes. It decreased the expression of the BCl-2 gene, which is an anti-apoptotic gene [18]. Rania et al., who studied the effect of ciprofloxacin derivatives on colorectal cancer (HCT-116) and non-small lung carcinoma (A549) cells, found similar results. Ciprofloxacin derivatives were found to have an anti-proliferative effect on both cell lines by increasing the expression of p53 and Bax proteins while decreasing the expression of p21 and bcl2 [34].

According to this finding, this newly synthesized (N-4 piperazine 4-chlorophenyl chalcone using urea as a linker) CP derivative induces G2/M phase cell cycle arrest and apoptosis by up-regulating Bax and Caspase 9 expression while down-regulating Bcl-2 expression. The findings of this investigation may shed new light on the therapeutic capabilities of this new ciprofloxacin derivative for treating colorectal cancer (HCT-116). A fuller understanding of the chemical mechanism underlying this derivative’s anti-proliferative activity would require more investigation.

## 4. Experimental

### 4.1. Chemistry

All reactions were monitored by TLC using Merck (Boston, MA, USA) 9385 pre-coated aluminum plate silica gel (Kieselgel 60) 5 cm × 20 cm plates with a layer thickness of 0.2 mm. The spots were detected by exposure to UV-lamp at λ = 254 nm. Melting points were determined on Stuart’s electrothermal melting point apparatus and were uncorrected. NMR spectra (400 MHz for ^1^H) were observed in DMSO-*d*6 on a Bruker AM400 spectrometer with tetramethyl silane as the internal standard. Chemical shift values are given in parts per million (ppm) using DMSO-d6 as a solvent, and coupling constants are designated as (*J*) in Hz. Splitting patterns are designated as follows: s, singlet; d, doublet; dd, doublet of doublet; t, triplet; q, quartet; m, multiplet; brs, broad singlet.

#### 7-(4-((4-(3-(4-Chlorophenyl) acryloyl) phenyl) carbamoyl) piperazin-1-yl)-1-cyclopropyl-6-fluoro-4-oxo-1,4-dihydroquinoline-3-carboxylic acid (CP derivative)

A 0.98 g (0.002 mol) of ciprofloxacin derivative was mixed with an equal amount of p-chlorobenzaldehyde (0.0021 mol) in 40 mL of absolute ethanol. Then, 0.25 mol of NaOH (60% *w*/*v*) was added slowly. The reaction mixture was stirred in a chilled container filled with ice for 30 min and subsequently at ambient temperature until a solid substance was produced within 12 h. The product was separated by filtration and then rinsed extensively with chilled distilled water and methanol. The product underwent recrystallization using absolute ethanol [18].

Yellow powder; 0.83 g, 66.0% yield; mp: 272–273 °C, reported 271–273 °C [18]; ^1^H NMR (400 MHz, DMSO-*d*6) δ = 1.18–1.22 (2H, m, cyclopropyl-H), 1.32–1.36 (2H, m, cyclopropyl-H), 3.39–3.44 (4H, m, piperazinyl-H), 3.78–3.87 (5H, m, 4-piperazinyl-H and cyclopropyl-H), 7.52 (2H, d, *J_H-H_* = 8.0 Hz, Ar-H), 7.56–7.69 (2H, m, =CH and Ar-H), 7.72 (2H, d, *J_H-F_* = 8.0 Hz, H-8-quinolone and Ar-H), 7.81–8.07 (4H, m, =CH, H-5-quinolone, and 2 Ar-H), 8.11 (2H, d, *J_H-H_* = 8.3 Hz, Ar-H), 8.68 (1H, s, H-2-quinolone), 9.06 (1H, br, CONH), 15.12 (1H, brs, carboxylic-H).

### 4.2. Biology

#### 4.2.1. Materials and Methods

##### Cell Culture

From the American Type Culture Collection (ATCC, Manassas, VA, USA), the HCT 116 cell line was obtained. The cells were grown in fresh Dulbecco’s Modified Eagle’s Medium (DMEM, Sigma-Aldrich, Inc., St Louis, MO, USA) with 10% fetal bovine serum (FBS, BioSolutions International, Melbourne, Australia), 1% penicillin-streptomycin mixture (Invitrogen, Grand Island, NY, USA), and 1% L-glutamine (Sigma-Aldrich, Inc.) in a humidified 5% CO_2_ atmosphere at 37 °C.

#### 4.2.2. In Vitro Screening of the Anticancer Activity at a Single Dose of 10 μM

In brief, the primary anticancer assay was conducted on a panel of approximately 60 human tumor cell lines representing nine types of cancer, following the protocol set by the Drug Evaluation Branch at the National Cancer Institute, Bethesda. The CP derivative was screened at a single concentration (10^−5^ M), with cultures incubated for 48 h. Cell growth was then measured using SRB, a protein-binding dye, and results were reported as the percentage growth of treated cells relative to untreated controls. Spectrophotometric measurements were taken to determine the growth percentage compared to untreated samples [16].

#### 4.2.3. Cell Viability Assay

The 3-(4,5-dimethylthiazol-2-yl)-2,5-diphenyltetrazolium bromide (MTT) assay is an effective method for assessing in vitro cytotoxicity in multiwell plates. This technique evaluates cell growth based on the ability of viable cells to convert the yellow MTT compound into a blue formazan product through a mitochondrial reduction process. HCT 116 cells were incubated in 96-well plates for 24 h. The quantity of viable cells, both with and without the test compounds (control), correlates with the intensity of the blue color, which was quantified spectrophotometrically at a wavelength of 570 nm using a ROBONIK (Ambernath, India) P2000 spectrophotometer. Background absorbance at 690 nm was measured and subtracted from the 570 nm readings. Each compound was tested at five concentrations, ranging from 0.39 μM to 100 μM in semi-logarithmic dilution. All experiments were conducted in triplicate, and IC_50_ values (the concentration at which cell viability is inhibited by 50%) were determined using sigmoidal dose-response curve models [1].

#### 4.2.4. Annexin V Assay

Apoptosis was assessed using the Annexin V-FITC apoptosis detection kit, following the manufacturer’s protocol (Sigma-Aldrich, Eschenstr, Taufkirchen, Germany). This kit, used in conjunction with flow cytometry, facilitates the quantification of apoptotic cells. By employing Annexin V/PI double staining, the method enables the detection of phosphatidylserine translocation to the outer leaflet of the cell membrane. Annexin V binds to phosphatidylserine in cells undergoing late apoptosis, but due to compromised membrane integrity, these can be differentiated from early apoptotic cells using PI staining. A total of 1 × 10^5^ cells were incubated with the CP derivative for 24 h, after which they were collected in Falcon tubes. The cells were washed twice with PBS after centrifugation, resuspended in binding buffer, and treated with Annexin V (5 µL) and PI (10 µL) in a 500 µL cell suspension. This mixture was incubated for 30 min at 4 °C in the dark. The percentage of apoptotic cells was determined by analyzing 10,000 cells per sample on a FACS Calibur flow cytometer [18].

#### 4.2.5. Cell Cycle Analysis

HCT 116 cells were exposed to CP derivative at its IC_50_ concentration for 24 h. Following treatment, the cells were washed twice with ice-cold phosphate-buffered saline (PBS, pH = 7.4, Sigma-Aldrich, Inc.), collected by centrifugation, and fixed in ice-cold 66% ethanol. They were then washed with PBS, resuspended in a solution containing 0.1 mg/mL RNase for 30 min, stained with 50 µg/mL propidium iodide (PI), and analyzed by flow cytometry using the FACSCalibur system (Becton Dickinson, Franklin Lakes, NJ, USA). Cell cycle distributions were calculated using CellQuest software (Becton Dickinson). Treatment with CP derivative affected the normal cell cycle distribution in the HCT 116 cells, as observed in the analysis [1].

#### 4.2.6. A Isolation and Real-Time qPCR Assay

Cells were treated with the CP derivative at its IC_50_ concentration for 24 h, followed by RNA extraction using the Qiagen kit (Hilden, Germany) according to the manufacturer’s protocol. Gene expression of Bax, caspase 9, and Bcl-2 was analyzed through real-time quantitative PCR (qPCR) using the Rotor-Gene 6000 software, with β-actin as the reference and doxorubicin as a positive control [35,36,37]. Primer sequences are provided in Table 4, ensuring high specificity and efficiency. The one-step RT-PCR kit from Qiagen (Germantown, MD, USA) facilitated precise amplification under optimal conditions: 100 ng RNA, 1× buffer, 0.6 µM primers, 400 µM dNTPs, and 2 µL enzyme mix. Conditions included an initial incubation at 50 °C for 30 min, followed by 15 min at 95 °C, then 40 cycles (94 °C for 1 min, 55 °C for 1 min, 72 °C for 1 min), with a final 10 min extension at 72 °C. Each sample was tested in triplicate to ensure linear amplification and consistency. The cycle threshold (Ct) was determined and averaged across replicates. A melting curve analysis (60–95 °C) verified primer specificity, eliminating non-specific products and contamination. Target gene expression in treated cells was quantified relative to controls, normalized to β-actin levels [38,39,40].

#### 4.2.7. Topoisomerases I/II Inhibition Assay

According to reported (TopoGEN, Inc., Buena Vista, CO, USA) (Inspiralis Limited, Norwich, UK) protocols, the topoisomerase inhibition assays for human DNA topoisomerase I and II were performed using a selected Cp derivative, with topotecan and etoposide as comparators. For the Topo I assay, a commercial kit provided necessary reagents, including supercoiled DNA substrate and a 10× assay buffer. The reaction mixture, comprising distilled water, assay buffer, DNA substrate, and test compound, was incubated with Topo I enzyme, and reactions were terminated with stop buffer before running on a 1% agarose gel to visualize enzyme activity through changes in supercoiled DNA bands. For the Topo II assay, pBR322 plasmid served as the substrate, and the reaction mix included Topo II buffer, dilution buffer, ATP, and the test compounds, with reactions stopped by adding STEB and chloroform/isoamyl alcohol. The effectiveness of inhibitors like etoposide was assessed through gel electrophoresis and IC_50_ determination, alongside a colorimetric assay using biotin-conjugated antibodies and HRP-avidin, with absorbance measured at 450 nm [41].

### 4.3. In Silico Studies

#### 4.3.1. Docking Studies

Molecular docking was performed using Auto Dock Vina, and Free Discovery Studio Visualizer was utilized to visualize the optimal docking poses. CP derivative, in comparison to topotecan and etoposide, was docked into the active sites of topo I (PDB ID: 1K4T) and topo II (PDB: 7YQ8), respectively, to suggest a possible mechanism of action and obtain possible binding modes between the CP derivative and the active sites of both topo I and topo II. The molecular structure of the CP derivative was created and refined using the chemical structure drawing software Marvin Sketch (Marvin JS, accessed on 24 August 2024) and the molecular modeling program Avogadro. The structures of human topo I (PDB ID: 1K4T) and topo II (PDB ID: 7YQ8) were obtained from the Protein Data Bank. The co-crystallized ligands were removed from the protein using Autodock techniques, and then Kollman charges and polar hydrogens were added. The *x*, *y*, and *z* axes’ grid coordinates for topo I were set to 22.1960, 0.8988, and 30.4129 with grid sizes of 61, 66, and 61, respectively, and for topo II were set to 116.953, 127.529, and 128.491 with grid sizes of 25.9567, 11.4206, and 19.3211, respectively [12].

#### 4.3.2. Physicochemical and Pharmacokinetic Prediction

The physicochemical characteristics and pharmacokinetics of the CP derivative were forecasted using Swiss ADME, a freely accessible service offered by the Swiss Institute of Bioinformatics [42]. The BOILED Egg graph illustrates the correlation between TPSA and WLOGP. The white region represents the highest probability of gastrointestinal absorption, while the yolk region indicates the greatest likelihood of BBB permeability. Lipophilicity is measured using the consensus log Po/w, which SwissADME calculates. The number represents the mean of five log P values estimated by different publicly accessible models, specifically XLOGP3, MLOGP, SILICOS-IT, iLOGP, and their proprietary model WLOGP, which is also employed in the BOILED Egg plot. The bioavailability radar consists of six specific physicochemical properties: lipophilicity (ranging from −0.7 to +5.0 for XLOGP3), size (ranging from 150 g/mol to 500 g/mol for MV), polarity (ranging from 20 Å2 to 130 Å2 for TPSA), insolubility (ranging from 0 to 6 for Log S (ESOL)), unsaturation (ranging from 0.25 to 1.0 for Fraction Csp3), and flexibility (ranging from 0 to more than 9 for the number of rotatable bonds). The central pink hexagon represents the optimal range for all six criteria. The Lipinski filter is utilized to assess the drug-like characteristics of synthesized substances. The evaluation considers the following criteria: The molecular weight (MW) must not exceed 500. The MLOGP value, which measures lipophilicity, must not exceed 4.15. The number of nitrogen or oxygen atoms must not exceed 10, and the number of NH or OH groups must not exceed 5.

## 5. Conclusions

In conclusion, this study explores the anticancer potential of a new ciprofloxacin derivative, demonstrating its inhibitory effects on topoisomerases I and II and its ability to induce apoptosis in colorectal cancer cells. It showed remarkable potency against HCT-116 and LOX IMVI cancer cell lines, with higher efficacy than the standard drug, Staurosporine. Mechanistic studies revealed that the new ciprofloxacin derivative promotes apoptosis by upregulating pro-apoptotic genes (Bax and Caspase 9) and downregulating the anti-apoptotic gene (Bcl-2). Additionally, it causes cell cycle arrest at the G2/M phase, which further contributes to its antiproliferative effects. Molecular docking studies supported these findings by demonstrating strong binding affinities of the compound to the active sites of Topoisomerases I and II. These results suggest that the ciprofloxacin-chalcone hybrid could be a promising lead compound for developing new anticancer therapies, potentially advancing cancer treatment with reduced cytotoxicity toward normal cells. Further optimization and in vivo studies are warranted to explore its therapeutic potential fully.

## Figures and Tables

**Table 1:** Topoisomerase I/II inhibitory activity by Cp derivative, topotecan, and etoposide measured as IC_50_ in µM ± SEM.

Compound	IC_50_ in µM	
	Topoisomerase I	Topoisomerase II
**Cp derivative**	37.5	19.9
**Topotecan**	23.0	ND
**Etoposide**	ND	28.9

ND: Not determined.

**Table 2:** Molecular docking data for CP derivative, topotecan, and etoposide against human Topo I (PDB: ID 1K4T) and Topo IIβ (PDB: ID 7YQ8).

Compound	Target	Binding Affinity (Kcal/mol)	Amino Acid Residue DNA Nucleotide Base	Types of Interaction
**Topotecan**	Topo I	−10.26	LYS 532 ASP 533 ARG 364 GLU 365 DC 112 DA 113 TGP 11 DT 10	H-bond H-bond H-bond Pi-Anion Pi-Pi Pi-Pi Pi-Pi Pi-Pi
**CP derivative**	Topo I	−12.04	TRP 416	H-bond
			ASN 419	H-bond
			ARG 375	Pi-Anion
			DG 112	Pi-Alkyl
			LYS 425	Pi-Alkyl
			GLU 356	Halogen
			ASP 533	Pi-Anion
			DA 113	Pi-Pi
			TGP 11	Pi-Pi
			DT 10	Pi-Pi
			HIS 632	Pi-Alkyl
			ILE 535	Pi-Alkyl
			ILE 420	Pi-Alkyl
			LYS 374	Pi-Alkyl
			PHE 361	Pi-Alkyl
**Etoposide**	Topo IIβ	−8.62	DC 28 DG 27 DG 23 ARG 508 PRO 824 MET 787	H-bond Pi-Pi Pi-Pi Pi-cation Pi-Alkyl Pi-Alkyl
**CP derivative**	Topo IIβ	−8.01	GLY 638 SER 485 GLU 482 GLY 483 PRO 824 ASP 562 DC 28 DG 27 DG 323	H-bond H-bond Halogen Halogen Pi-Alkyl Negative-Negative H-bond Pi-Pi Pi-Pi

**Table 3:** Physicochemical properties of target compound **CP derivative**.

**CP derivative**	**Physicochemical Properties of CP Derivative**							
	Heavy atoms	Aromatic heavy atoms	Fraction Csp3	Rotatable bonds	HBAs	HBDs	MR	TPSA
	44	22	0.21	9	6	2	174.38	111.95
	**Lipophilicity Parameters of CP Derivative**							
	iLOGP	XLOGP3	WLOGP	MLOGP	Silicos-IT Log P	Consensus Log P		
	3.18	4.91	5.37	3.51	4.79	4.35		
	**Water Solubility Parameters of CP Derivative**							
	ESOL Solubility (mg/mL)	ESOL Class	Ali Solubility (mg/mL)	Ali Class	Silicos-IT Solubility (mg/mL)	Silicos-IT class		
	1.85 × 10^−4^	Poorly Soluble	6.19 × 10^−5^	Poorly Soluble	4.04 × 10^−6^	Poorly Soluble		
	**Pharmacokinetics of CP Derivative**							
	GI absorption	BBB permeant	Pgp substrate	CYP1A2 inhibitor	CYP1A4 inhibitor	CYP2C9 inhibitor	CYP2D6 inhibitor	log Kp (cm/s)
	low	no	no	no	no	yes	no	−6.57
	**Drug Likeness Parameters of CP Derivative.**							
	Lipinski violations	Ghose violations	Veber violations	Egan violations	Muegge violations	Bioavailability Score		
	1	3	0	0	1	0.56		

**Table 4:** Primers used in real-time qPCR assay.

Primer	Sequence of Primer
Bax	F 5′-ATGTTTTCTGACGGCAACTTC -3′ R 5′- AGTCCAATGTCCAGCCCAT-3′
BCL-2	F 5′-ATGTGTGTGGAGACCGTCAA -3′ R 5′-GCCGTACAGTTCCACAAAGG -3′
Caspase-9	F 5′-TCA GTG ACG TCT GTG TTC AGG AGA-3′ R 5′-TTG TTG ATG ATG AGG CAG TAG CCG-3′
β-Actin	F 5′-GTGACATCCACACCCAGAGG-3′ R 5′-ACAGGATGTCAAAACTGCCC-3′

## Data Availability

The original contributions presented in this study are included in the article and Supplementary Materials; further inquiries can be directed to the corresponding author.

## Supplementary Materials

The following supporting information can be downloaded at: https://www.mdpi.com/article/10.3390/molecules29225382/s1.

## References

[1] Abdel-Rahman I.M., Mustafa M., Mohamed S.A., Yahia R., Abdel-Aziz M., Abuo-Rahma G.E.-D.A., Hayallah A.M. (2021). Novel Mannich bases of ciprofloxacin with improved physicochemical properties, antibacterial, anticancer activities and caspase-3 mediated apoptosis. Bioorganic Chem..

[2] Mohammad R.M., Muqbil I., Lowe L., Yedjou C., Hsu H.Y., Lin L.T., Siegelin M.D., Fimognari C., Kumar N.B., Dou Q.P. (2015). Broad targeting of resistance to apoptosis in cancer. Seminars in Cancer Biology.

[3] Radwan M.O., Sakai S., Hassan A.N., Uesugi M., Sakamoto M., Toma T., Abourehab M.A.S., Badran M.M., Tateishi H., Nishimura N. (2024). Discovery of cytotoxic truncated vitamin D derivatives against both bortezomib-sensitive and bortezomib-resistant multiple myeloma phenotypes. Med. Chem. Res..

[4] McClendon A.K., Osheroff N. (2007). DNA topoisomerase II, genotoxicity, and cancer. Mutat. Res./Fundam. Mol. Mech. Mutagen..

[5] Ansari M., Shokrzadeh M., Karima S., Rajaei S., Fallah M., Ghassemi-Barghi N., Ghasemian M., Emami S. (2020). New thiazole-2(3H)-thiones containing 4-(3,4,5-trimethoxyphenyl) moiety as anticancer agents. Eur. J. Med. Chem..

[6] El-Metwally S.A., Khalil A.K., El-Sayed W.M. (2020). Design, molecular modeling and anticancer evaluation of thieno[2,3-d]pyrimidine derivatives as inhibitors of topoisomerase II. Bioorganic Chem..

[7] Mohammed H.H.H., Ali D.M.E., Badr M., Habib A.G.K., Mahmoud A.M., Farhan S.M., Gany S.S.H.A.E., Mohamad S.A., Hayallah A.M., Abbas S.H. (2023). Synthesis and molecular docking of new N4-piperazinyl ciprofloxacin hybrids as antimicrobial DNA gyrase inhibitors. Mol. Divers..

[8] Delgado J.L., Hsieh C.-M., Chan N.-L., Hiasa H. (2018). Topoisomerases as Anticancer Targets. Biochem. J..

[9] Ahadi H., Emami S. (2020). Modification of 7-piperazinylquinolone antibacterials to promising anticancer lead compounds: Synthesis and in vitro studies. Eur. J. Med. Chem..

[10] Alhaj-Suliman S.O., Naguib Y.W., Wafa E.I., Saha S., Ebeid K., Meng X., Mohammed H.H., Abuo-Rahma G.E.-D.A., Yang S., Salem A.K. (2023). A ciprofloxacin derivative with four mechanisms of action overcomes paclitaxel resistance in p53-mutant and MDR1 gene-expressing type II human endometrial cancer. Biomaterials.

[11] Tillotson G.S. (1996). Quinolones: Structure-activity relationships and future predictions. J. Med. Microbiol..

[12] Hashem H., Hassan A., Abdelmagid W.M., Habib A.G.K., Abdel-Aal M.A.A., Elshamsy A.M., El Zawily A., Radwan I.T., Bräse S., Abdel-Samea A.S. (2024). Synthesis of New Thiazole-Privileged Chalcones as Tubulin Polymerization Inhibitors with Potential Anticancer Activities. Pharmaceuticals.

[13] Shukla S., Sood A.K., Goyal K., Singh A., Sharma V., Guliya N., Gulati S., Kumar S. (2021). Chalcone scaffolds as anticancer drugs: A review on molecular insight in action of mechanisms and anticancer properties. Anti-Cancer Agents Med. Chem. (Former. Curr. Med. Chem. -Anti-Cancer Agents).

[14] Swedan H.K., Kassab A.E., Gedawy E.M., Elmeligie S.E. (2023). Design, synthesis, and biological evaluation of novel ciprofloxacin derivatives as potential anticancer agents targeting topoisomerase II enzyme. J. Enzym. Inhib. Med. Chem..

[15] Struga M., Roszkowski P., Bielenica A., Otto-Ślusarczyk D., Stępień K., Stefańska J., Zabost A., Augustynowicz-Kopeć E., Koliński M., Kmiecik S. (2023). N-Acylated ciprofloxacin derivatives: Synthesis and in vitro biological evaluation as antibacterial and anticancer agents. ACS Omega.

[16] Mohammed H.H.H., Abd El-Hafeez A.A., Abbas S.H., Abdelhafez E.-S.M.N., Abuo-Rahma G.E.-D.A. (2016). New antiproliferative 7-(4-(N-substituted carbamoylmethyl)piperazin-1-yl) derivatives of ciprofloxacin induce cell cycle arrest at G2/M phase. Bioorg. Med. Chem..

[17] Al-Taweel S., Al-Saraireh Y., Al-Trawneh S., Alshahateet S., Al-Tarawneh R., Ayed N., Alkhojah M., Wisam A.K., Zereini W., Al-Qaralleh O. (2023). Synthesis and biological evaluation of ciprofloxacin–1, 2, 3-triazole hybrids as antitumor, antibacterial, and antioxidant agents. Heliyon.

[18] Mohammed H.H.H., Abbas S.H., Hayallah A.M., Abuo-Rahma G.E.-D.A., Mostafa Y.A. (2021). Novel urea linked ciprofloxacin-chalcone hybrids having antiproliferative topoisomerases I/II inhibitory activities and caspases-mediated apoptosis. Bioorganic Chem..

[19] Ghosh A.K., Brindisi M. (2019). Urea derivatives in modern drug discovery and medicinal chemistry. J. Med. Chem..

[20] Kaina B. (2003). DNA damage-triggered apoptosis: Critical role of DNA repair, double-strand breaks, cell proliferation and signaling. Biochem. Pharmacol..

[21] Pu L., Amoscato A.A., Bier M.E., Lazo J.S. (2002). Dual G1 and G2 phase inhibition by a novel, selective Cdc25 inhibitor 6-chloro-7-[corrected](2-morpholin-4-ylethylamino)-quinoline- 5,8-dione. J. Biol. Chem..

[22] Hawtin R.E., Stockett D.E., Byl J.A.W., McDowell R.S., Nguyen T., Arkin M.R., Conroy A., Yang W., Osheroff N., Fox J.A. (2010). Voreloxin is an anticancer quinolone derivative that intercalates DNA and poisons topoisomerase II. PLoS ONE.

[23] Yadav V., Varshney P., Sultana S., Yadav J., Saini N. (2015). Moxifloxacin and ciprofloxacin induces S-phase arrest and augments apoptotic effects of cisplatin in human pancreatic cancer cells via ERK activation. BMC Cancer.

[24] Samir M., Ramadan M., Abdelrahman M.H., Abdelbaset M.S., Abourehab M.A.S., Abdel-Aziz M., Abuo-Rahma G.E.-D.A. (2021). 3,7-bis-benzylidene hydrazide ciprofloxacin derivatives as promising antiproliferative dual TOP I & TOP II isomerases inhibitors. Bioorganic Chem..

[25] Staker B.L., Hjerrild K., Feese M.D., Behnke C.A., Burgin A.B., Stewart L. (2002). The mechanism of topoisomerase I poisoning by a camptothecin analog. Proc. Natl. Acad. Sci. USA.

[26] Bunch H., Kim D., Naganuma M., Nakagawa R., Cong A., Jeong J., Ehara H., Vu H., Chang J.H., Schellenberg M.J. (2023). ERK2-topoisomerase II regulatory axis is important for gene activation in immediate early genes. Nat. Commun..

[27] Talevi A. (2015). Multi-target pharmacology: Possibilities and limitations of the “skeleton key approach” from a medicinal chemist perspective. Front. Pharmacol..

[28] Yadav V., Talwar P. (2019). Repositioning of fluoroquinolones from antibiotic to anti-cancer agents: An underestimated truth. Biomed. Pharmacother..

[29] Brody H. (2015). Colorectal cancer. Nature.

[30] Liang H.X., Yu Y.H., Li X.H., Tang N.F., Hu G.Q., Liu B. (2019). Apoptosis of human hepatocellular carcinoma cells SMMC-7721 induced by C-3 methylidene thiazolidinedione acetic acid. Int. J. Clin. Exp. Med..

[31] Aziz H.A., El-Saghier A.M., Badr M., Elsadek B.E.M., Abuo-Rahma G.E.-D.A., Shoman M.E. (2024). Design, synthesis and mechanistic study of N-4-Piperazinyl Butyryl Thiazolidinedione derivatives of ciprofloxacin with Anticancer Activity via Topoisomerase I/II inhibition. Sci. Rep..

[32] Singh R., Letai A., Sarosiek K. (2019). Regulation of apoptosis in health and disease: The balancing act of BCL-2 family proteins. Nat. Rev. Mol. Cell Biol..

[33] Pfeffer C.M., Singh A.T. (2018). Apoptosis: A target for anticancer therapy. Int. J. Mol. Sci..

[34] Alaaeldin R., Nazmy M.H., Abdel-Aziz M., Abuo-Rahma G.E.D.A., Fathy M. (2020). Cell Cycle Arrest and Apoptotic Effect of 7-(4-(N-substituted carbamoylmethyl) piperazin-1-yl) Ciprofloxacin-derivative on HCT 116 and A549 Cancer Cells. Anticancer. Res..

[35] Mohamed M.S., Abdelhamid A.O., Almutairi F.M., Ali A.G., Bishr M.K. (2018). Induction of apoptosis by pyrazolo[3,4-d]pyridazine derivative in lung cancer cells via disruption of bcl-2/bax expression balance. Bioorganic Med. Chem..

[36] Shahat A.S. (2017). Antioxidant and anticancer activities of yeast grown on commercial media. Int. J. Biol. Chem. Sci..

[37] Bas A., Forsberg G., Hammarstrom S., Hammarstrom M.L. (2004). Utility of the housekeeping genes 18s rrna, beta-actin and glyceraldehyde-3-phosphate-dehydrogenase for normalization in real-time quantitative reverse transcriptase-polymerase chain reaction analysis of gene expression in human t lymphocytes. Scand. J. Immunol..

[38] Hinz B., Ramer R., Eichele K., Weinzierl U., Brune K. (2004). Up-regulation of cyclooxygenase-2 expression is involved in r(+)- methanandamide-induced apoptotic death of human neuroglioma cells. Mol. Pharmacol..

[39] Kleiboeker S.B. (2003). Applications of competitor RNA in diagnostic reverse transcription-pcr. J. Clin. Microbiol..

[40] Cha S.H., Chang C.C., Yoon K.J. (2004). Instability of the restriction fragment length polymorphism pattern of open reading frame 5 of porcine reproductive and respiratory syndrome virus during sequential pig-to-pig passages. J. Clin. Microbiol..

[41] Trask D.K., DiDonato J.A., Muller M.T. (1984). Rapid detection and isolation of covalent DNA/protein complexes: Application to topoisomerase I and II. EMBO J..

[42] Hassan A., Mubarak F.A.F., Shehadi I.A., Mosallam A.M., Temairk H., Badr M., Abdelmonsef A.H. (2023). Design and biological evaluation of 3-substituted quinazoline-2,4(1H,3H)-dione derivatives as dual c-Met/VEGFR-2-TK inhibitors. J. Enz. Inhib. Med. Chem..

