# Supplementary material for: Unveiling the Anticancer Potential of a New Ciprofloxacin-Chalcone Hybrid as an Inhibitor of Topoisomerases I & II and Apoptotic Inducer
Source: Molecules. 2024 Nov 15;29(22):5382. doi: 10.3390/molecules29225382 (PMC11596536; doi:10.3390/molecules29225382)
Supplement: Supplementary file 1 [file molecules-29-05382-s001.zip › molecules-3205896-supplementary.pdf]

Supporting information

Unveiling the anticancer potential of a new ciprofloxacin-chalcone hybrid as an inhibitor of topoisomerases I & II and apoptotic inducer

Doaa Mohamed Elroby Ali[1†], Hossameldin A. Aziz[2†], Stefan Bräse [3]*, Areej Al Bahir[4], Abdullah Alkhammash[5], Gamal El-Din A. Abuo-Rahma[6,7], Ali M. Elshamsy[7], Hamada Hashem*[8], Walid M. Abdelmagid[9]

[1]Department of Biochemistry, Faculty of Pharmacy, Sohag University, Sohag, 82524, Egypt.

[2]Department of Pharmaceutical Chemistry, Faculty of Pharmacy, New Valley University, New Valley, 72511, Egypt.

[3]Institute of Biological and Chemical Systems – Functional Molecular Systems (IBCS-FMS), Karlsruhe Institute of Technology (KIT), Kaiserstrasse 12, 76131 Karlsruhe

[4]Chemistry Department, Faculty of Science, King Khalid University, Abha 64734, Saudi Arabia

[5]Department of Pharmacology, College of Pharmacy, Shaqra University, Shaqra, 11961, Saudi Arabia

[6]Department of Medicinal Chemistry, Faculty of Pharmacy, Minia University, Minia 61519, Egypt.

[7]Department of Pharmaceutical Chemistry, Faculty of Pharmacy, Deraya University, New Minia City, 61768, Egypt.

[8]Pharmaceutical Chemistry Department, Faculty of Pharmacy, Sohag University, Sohag, 82524, Egypt

[9]Medicinal Chemistry and Drug Discovery Research Centre, Swenam College, 210-6125 Sussex Avenue, Burnaby, BC, V5H 4G1, Canada

[†]Both authors contributed equally to this work.

[*]Correspondence: hamada.hashem@pharm.sohag.edu.eg (H.H.); stefan.braese@kit.edu (S.B.)

Contents

1. Chemistry 3

Figure S1: 1HNMR spectrum of compound CP-derivative (400 MHz, CDCl3) 3

2.Biology 4

2.1.Screening of the anticancer activity at 10 M 4

Figure S2: In vitro screening of the anticancer activity at single dose of 10 µM 5

2.2.Cell viability Assay 5

Table S1. Cytotoxicity results of CP-derivative in comparison to Staurosporine as a positive control against HCT-116 cancer cell line

2.3.Annexin V assay and cell cycle analysis using flow cytometry 5

Table S2. Cell cycle phase distribution of untreated cells and cells treated with the IC50 concentration of CP-derivative for 24 h. 5

Table S3. The apoptosis and necrosis assay of colon HCT-116 induced by DMSO (control, A), and CP-derivative (IC50, 4.98 M, B). 5

Figure S3. Cell cycle analysis and apoptosis of untreated HCT-116. 6

Figure S4. Cell cycle analysis and apoptosis of HCT-116 treated with IC50 of CP-derivative 6

2.4 Expression of Bax, Bcl-2 and Caspase 9 genes 7

Table S4. Expression of Bax, Bcl-2 and Caspase 9 genes (1) 7

Quantitation Report 7

Experiment Information 7

Table S5. Expression of Bax, Bcl-2 and Caspase 9 genes (2) 8

Figure S5. topoisomerase assay of CP-derivative versus topotecan 9

Figure S6. topoisomerase assay of CP-derivative versus etoposide 10

Figure S7. The superimposition of the redocked (**violet** color) and co-crystallized ligand (**blue** color) poses of etoposide 11

Figure S8. The superimposition of the redocked (**violet** color) and co-crystallized ligand (**blue** color) poses of topotecan 11

1. Chemistry

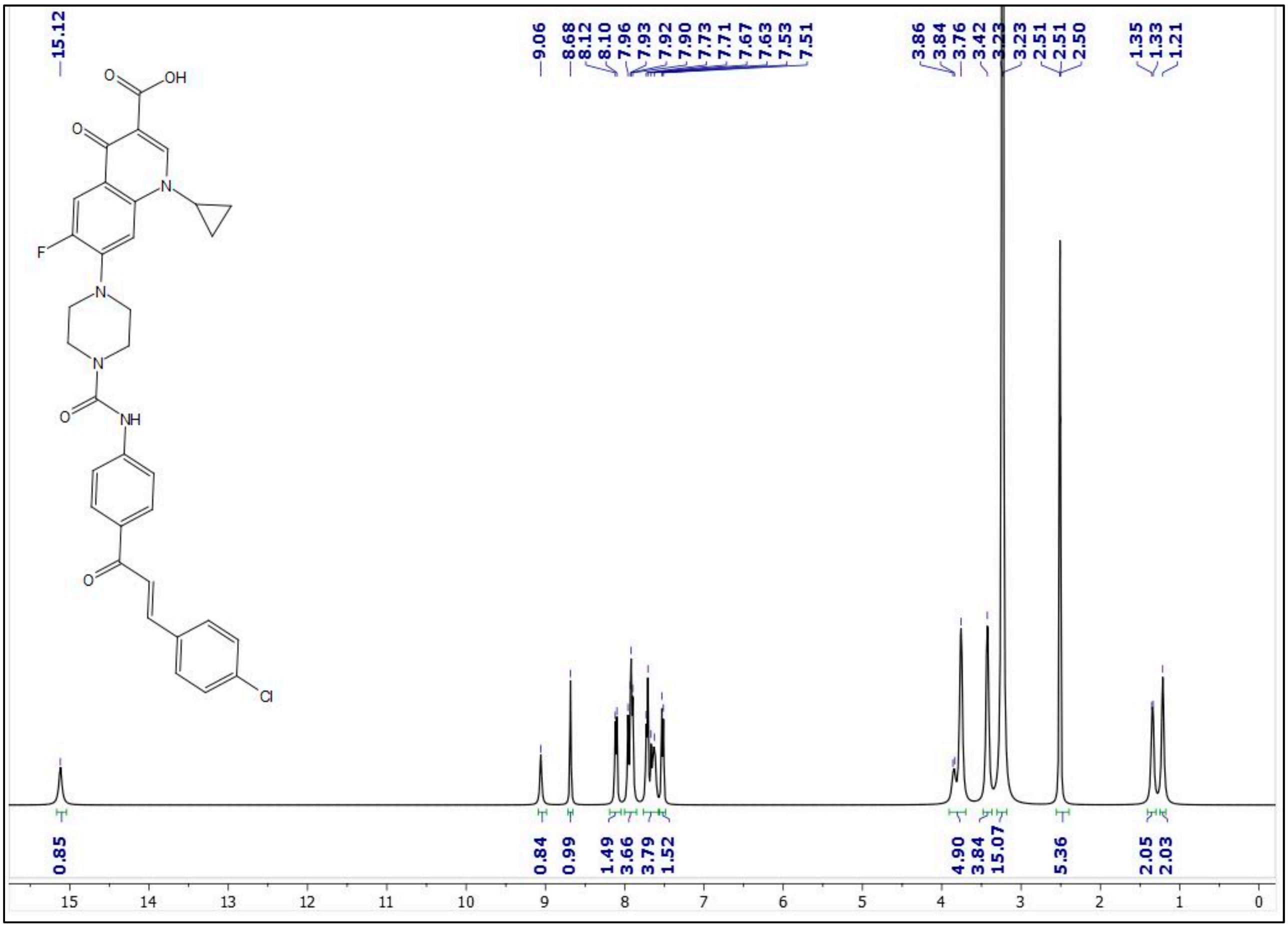

Figure S1: ^{1}HNMR spectrum of compound CP-derivative (400 MHz, $CDCl_3$)

2. Biology

2.1. Screening of the anticancer activity at 10 μM

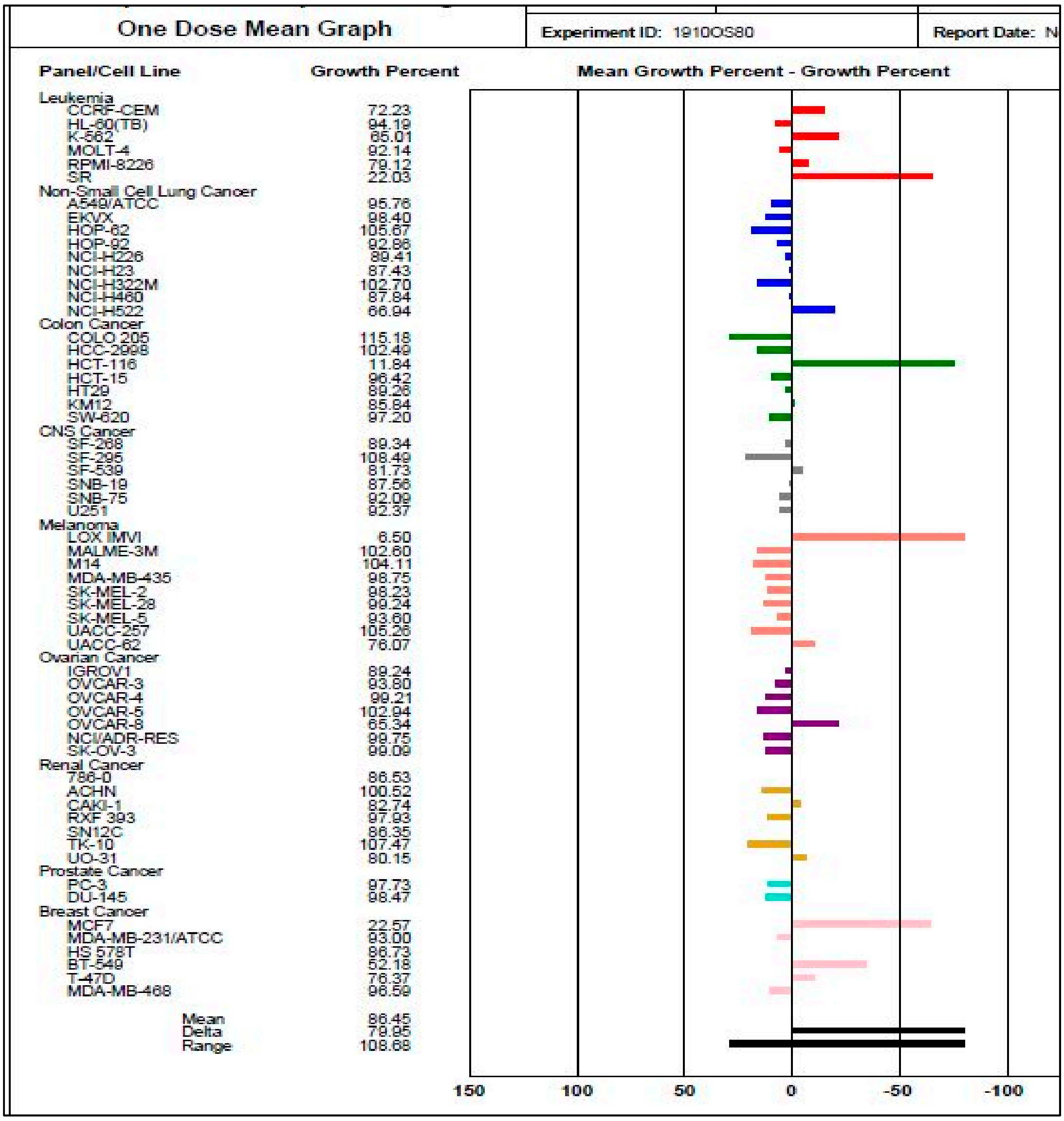

Figure S2: In vitro screening of the anticancer activity at single dose of 10 μM

2.2. Cell viability Assay

Table S1. Cytotoxicity results of CP-derivative in comparison to Staurosporine as a positive control against HCT-116 cancer cell line.

Ser	Sample		cytotoxicity IC50 uM		
	Code	M.W g/mol	HCT116	LOX IMVI	WI-38
1	CP-derivative		4.98±0.27	1.26 ± 0.02	15.96± 0.72
2	Staurosporine		8.29±0.39	1.63 ± 0.04	13.34 ± 0.54

2.3. Annexin V assay and cell cycle analysis using flowcytometry

Table S2. Cell cycle phase distribution of untreated cells and cells treated with the IC_{50} concentration of CP-derivative for 24 h.

ser	Sample data		Results DNA content				
	code	IC50 μM	%G0-G1	%S	%G2/M	%Pre-G1	Comment
1	CP-derivative		55.16	42.06	1.78	39.21	Cell growth arrest@ G2/M
2	control		51.36	39.42	9.22	1.99	

Table S3. The apoptosis and necrosis assay of colon HCT-116 induced by DMSO (control, A), and CP-derivative (IC_{50}, 4.98 μM, B).

s	code	Conc	Apoptosis			Necrosis
			Total	Early	Late	
1	CP-derivative		39.21	1.56	23.53	14.12
2	control		1.99	0.73	0.12	1.14

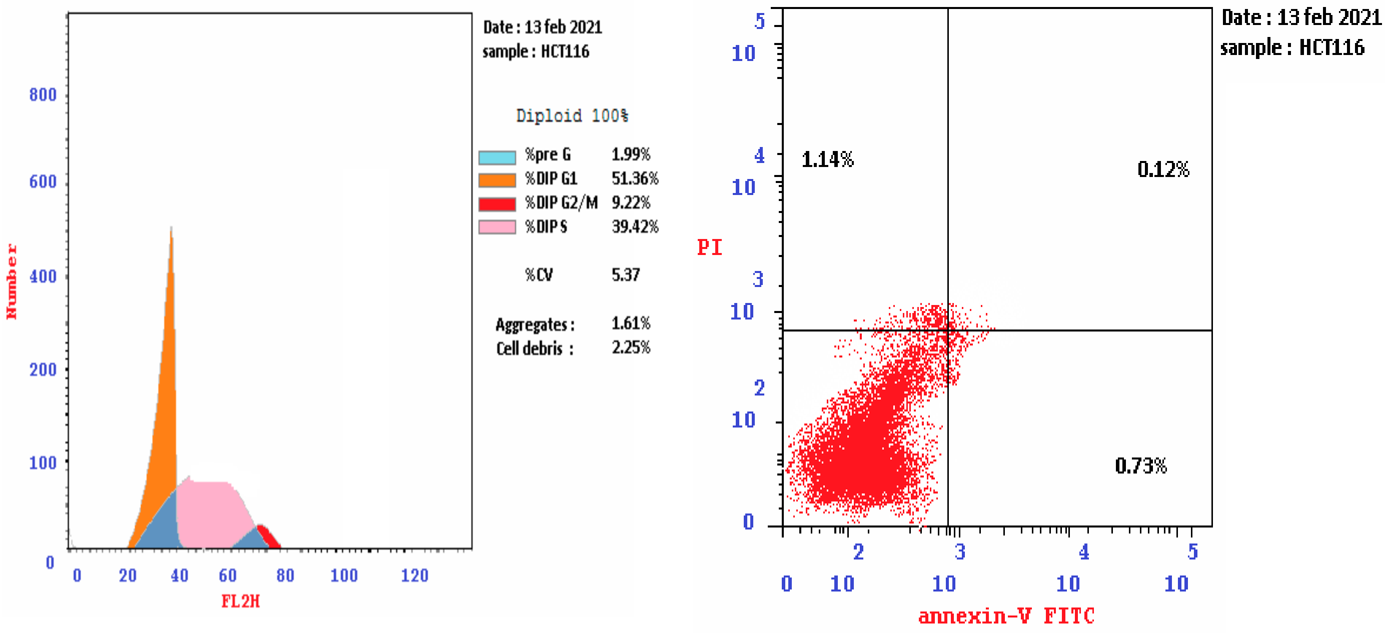

Figure S3. Cell cycle analysis and apoptosis of untreated HCT-116.

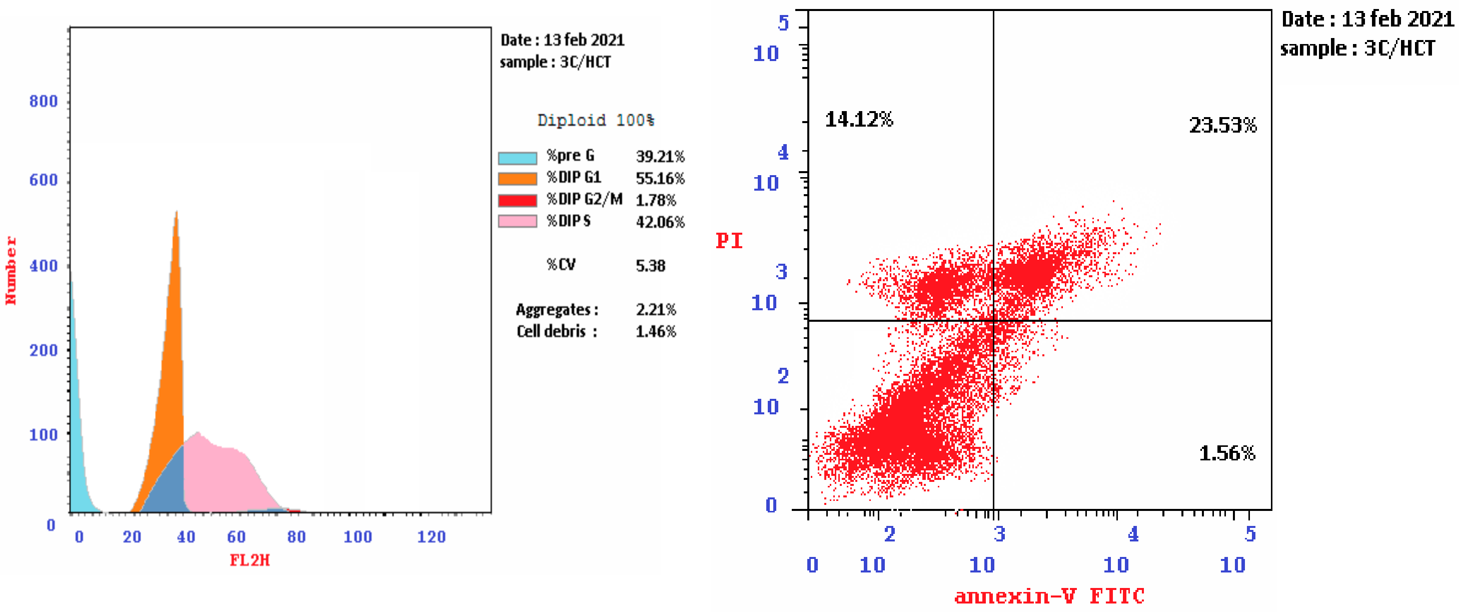

Figure S4. Cell cycle analysis and apoptosis of HCT-116 treated with IC50 of CP-derivative

2.4 Expression of Bax, Bcl-2 and Caspase 9 genes

Table S4. Expression of Bax, Bcl-2 and Caspase 9 genes

Ser	Sample			Gene Expression Fold Change			
	code	conc	Cells	Bax fld	Bcl2 fld	Casp9 fld	
	Cp-derivative		HCT116	4.614	0.320	5.573	
	control			1	1	1	

Detailed results

corbett RESEARCH
A Division of Corbett Life Science

14 Hilly Street Mortlake NSW 2137 Australia
T +61 2 9736 1320
F +61 2 9736 1364
W www.corbettlifescience.com

Quantitation Report

Experiment Information

Run Name	Run 2021-02-13 (1)
Run Start	2021-02-13 02:42:26 PM
Run Finish	2021-02-13 05:11:05 PM
Operator	ERA
Notes	---
Run On Software Version	Rotor-Gene 1.7.87
Run Signature	The Run Signature is valid.
Gain Green	10.
Gain Yellow	9.33

Quantitation data

This report generated by Rotor-Gene 6000 Series Software 1.7 (Build 87)
Copyright ©2000-2006 Corbett Research, a Division of Corbett Life Science. All rights reserved.
ISO 9001:2000 (Reg. No. QEC21313)

[redacted]

primers

CASP9-F 5'-TCA GTG ACG TCT GTG TTC AGG AGA-3'
CASP9-R 5'-TTG TTG ATG ATG AGG CAG TAG CCG-3'

[redacted]

Bax F 5'-ATGTTTTCTGACGGCAACTTC -3'
Bax R 5'- AGTCCAATGTCCAGCCCAT-3'

[redacted]

Bacl2 F 5'-ATGTGTGTGGAGACCGTCAA -3'
Bacl2 R 5'-GCCGTACAGTTCCACAAAGG -3'

[redacted]

β-actin F 5’-GTGACATCCACACCCAGAGG-3’

β-actin R 5’-ACAGGATGTCAAAACTGCCC-3’

Table S5. Expression of Bax, Bcl-2 and Caspase 9 genes

Sample			Bax							
			Control cells			Test cells				FLD
**Ser**	**code**	**Conc**	B Actin	Bax	ΔCTC	B Actin	Bax	ΔCTE	ΔΔ CT	2^ ΔΔCT
			HC	TC	TC-HC	HE	TE	TE-HE	ΔCTE-ΔCTC	Eamp=1.839
1	3C		23.51	33.49	9.98	23.88	31.35	7.47	-2.51	4.614239582
2	control		23.51	33.49	9.98	23.51	33.49	9.98	0	1

Sample			Bcl2							
			Control cells			Test cells				FLD
**Ser**	**code**	**Conc**	B Actin	Bcl2	ΔCTC	B Actin	Bcl2	ΔCTE	ΔΔ CT	2^ ΔΔCT
			HC	TC	TC-HC	HE	TE	TE-HE	ΔCTE-ΔCTC	Eamp=1.839
1	3C		23.51	28.94	5.43	23.88	31.18	7.3	1.87	0.320060564
2	control		23.51	28.94	5.43	23.51	28.94	5.43	0	1

Sample			Casp9							
			Control cells			Test cells				FLD
**Ser**	**code**	**Conc**	B Actin	Casp9	ΔCTC	B Actin	Casp9	ΔCTE	ΔΔ CT	2^ ΔΔCT
			HC	TC	TC-HC	HE	TE	TE-HE	ΔCTE-ΔCTC	Eamp=1.839
1	3C		23.51	32.91	9.4	23.88	30.46	6.58	-2.82	5.573403378
2	control		23.51	32.91	9.4	23.51	32.91	9.4	0	1

Topoisomerase I assay

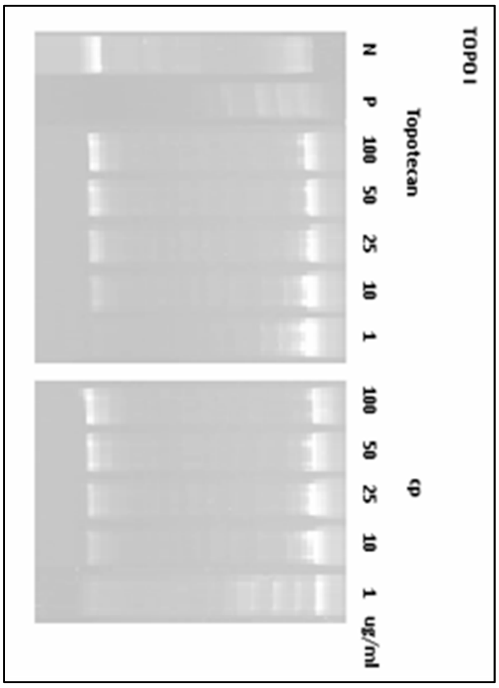

Figure S5. topoisomerase assay of CP-derivative versus topotecan

<u>Detailed results :</u>

TOPO I

code	IC50	conc	log	%inh
cp		100	2	81
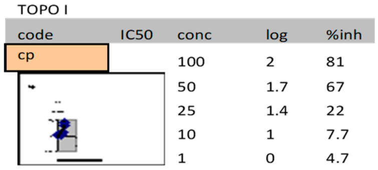		50	1.7	67
		25	1.4	22
		10	1	7.7
		1	0	4.7
EC				0
	[redacted]			

code	IC50	conc.uM	log conc	%inh
Topotecan		100	2	88
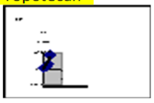		50	1.7	79
		25	1.4	31
		10	1	16
		1	0	7
EC				0
	[redacted]			

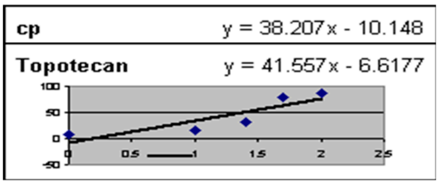

Topoisomerase II assay

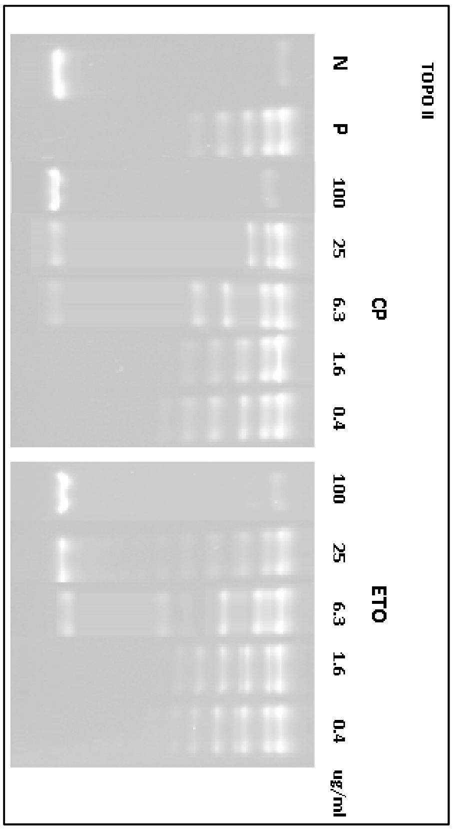

Figure S6. topoisomerase assay of CP-derivative versus topotecan

Detailed results:

**TOPO II**		[redacted]		
code	IC50	conc	log	%inh
cp		100	2	92
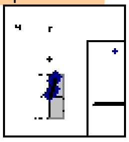		50	1.7	75
		25	1.4	38
		10	1	20
		1	0	7.6
EC				0
		[redacted]		[redacted]
code	IC50	conc	log	%inh
Etoposide		100	2	90
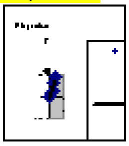		50	1.7	63
		25	1.4	28
		10	1	15
		1	0	3.8
EC				0
		[redacted]		[redacted]

Docking studies

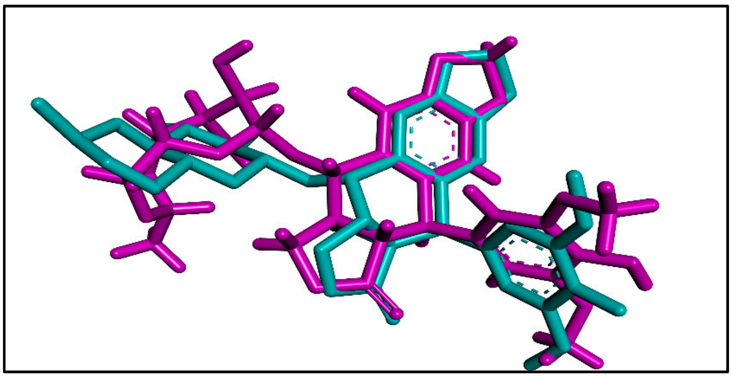

Figure S7. The superimposition of the redocked (**violet** color) and co-crystallized ligand (**blue** color) poses of etoposide.

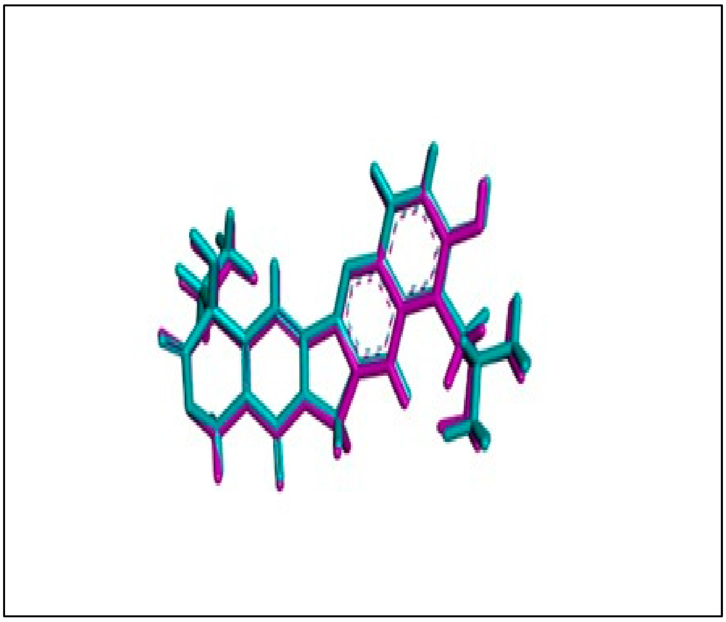

Figure S8. The superimposition of the redocked (**violet** color) and co-crystallized ligand (**blue** color) poses of Topotecan.
